# Effective and Safe Management of Oral Anticoagulation Therapy in Patients Who Use the Internet-Accessed Telecontrol Tool SintromacWeb

**DOI:** 10.2196/ijmr.3610

**Published:** 2015-04-21

**Authors:** Fernando Ferrando, Yolanda Mira

**Affiliations:** ^1^Hospital Universitari i Politècnic La FeUnidad de Hemostasia y TrombosisValenciaSpain

**Keywords:** oral anticoagulant therapy, International Normalized Ratio, Internet, self-management software, telecontrol, SintromacWeb

## Abstract

**Background:**

Despite the existing evidence that highlights the benefits of oral anticoagulation therapy (OAT) self-testing and self-management by patients in comparison with conventional control, significant progress is still needed in the implementation of computer-based, Internet-assisted systems for OAT within health care centers. The telecontrol tool “SintromacWeb” is a previously validated system for OAT management at home, which is currently operative and accessed by patients through a hospital Web portal.

**Objective:**

The intent of the study was to assess the effectiveness and safety of OAT management in patients using the SintromacWeb telecontrol system in reference to control in patients using the conventional system (management at the hematology department), in terms of time in therapeutic range (TTR) of International Normalized Ratio (INR).

**Methods:**

In this observational prospective study, patients were identified by their physician and divided in two groups according to the OAT management system that they were already using (conventional control or telecontrol with SintromacWeb). For 6 months, patients were required to visit the hematology department every time their physician considered it necessary according to usual clinical practice. Sociodemographic and clinical variables for the study were collected at first visit (baseline) and at those visits closest to 2, 4, and 6 months after first visit.

**Results:**

A total of 173 patients were evaluated, 87 with conventional control and 86 with telecontrol. Follow-up time was a median of 6.3 (range 5.2-8.1) months. The average time of OAT treatment prior to enrollment was 9.2 (SD 6.4) years. Patients in the telecontrol group tested their INR a median of 21 (range 4-22) days versus a median of 35 (range 14-45) days in patients in the conventional control group (*P*<.001). TTR in the telecontrol group was 107 (SD 37) days versus 94 (SD 37) days in the conventional control group (an increase of 12.6%; *P*=.02). In all visits, the percentage of TTR was higher in the telecontrol group (at the third visit: 59% vs 48%; *P*=.01). Higher TTR (positive coefficient) was associated with patients under OAT telecontrol (*P*=.03). Under-anticoagulation (INR<1.5) and over-anticoagulation (INR>5) were observed in 34 (19.7%, 34/173) and 38 (22.0%, 38/173) patients, respectively (no differences between treatment groups). Seven thrombotic and/or bleeding events were serious, 12 were non-serious, and most of them (5 and 10, respectively) occurred in the conventional control group.

**Conclusions:**

In clinical practice, OAT management with the Internet-based tool SintromacWeb is effective and safe for those patients who are eligible for OAT telecontrol.

## Introduction

Oral anticoagulation therapy (OAT) with vitamin K antagonists effectively reduces the risk of thromboembolism in patients with hereditary or acquired thrombophilia, heart valve replacement, atrial fibrillation, and other conditions [1]. Studies carried out in Spain and Italy showed a prevalence for OAT of 1.32% and 0.81%, respectively, with atrial fibrillation as the main cause of an indication for anticoagulation therapy (47.1% and 45.6%, respectively) [2,3].

The INR (International Normalized Ratio) is a standardized number obtained by means of a laboratory test that determines the degree of anticoagulation level achieved by the vitamin K antagonist [4]. The goals of OAT are both preventing thromboembolism and minimizing the risk of bleeding complications by reaching and maintaining the INR within the appropriate range for each patient, depending on their disease [5]. To summarize the INR control over time, percent time in therapeutic range (TTR) of INR is used.

Monitoring of OAT patients is conventionally carried out in hospitals and primary care centers, which are in contact with the referring hematologist in specialized hospitals. However, there is extensive literature that supports effectiveness and safety of OAT self-management by patients at home and shows that self-management at home is similar or even more effective and safer than in conventional control [6-9]. Decentralized management not only lightens the burden in health centers, but also prompts fewer monitoring visits to specialized centers, resulting in more freedom for the patient and improved quality of life [10-12]. Furthermore, that the patient is provided with greater responsibility in measuring and dosing their own INR can increase awareness, commitment, and interest in the management of their disease.

According to biomedical literature, any new management model should demonstrate anticoagulation control levels over 60% for TTR of INR to be considered safe and be within at least 5 to 10% compared with routine monitoring to declare it as a better model [6-8]. Since TTR is a parameter strongly associated with the occurrence of clinical events, its use as a primary endpoint in clinical trials of anticoagulation is recommended [13]. Few randomized trials assessing TTR-based effectiveness and/or safety of OAT self-testing programs have been performed so far [14-20]. Importantly, no trial has tested a validated, already implemented system in a hospital for OAT telecontrol at home.

“SintromacWeb” is a new-generation, Internet-based system developed by Grifols (Barcelona, Spain) as an alternative tool to conventional OAT management. SintromacWeb has already been successfully tested and validated in terms of reliability, consistency, and patient satisfaction [21]. The system is currently operative in the *La Fe* University Hospital (Valencia, Spain), and it is accessed by patients through the hospital Web portal.

Designed as the natural extension of its predecessor [21], this study prospectively assessed both effectiveness, in terms of TTR, and safety of OAT management in patients using the SintromacWeb system in clinical practice, compared to patients using management at the hematology department, a system considered conventional.

## Methods

### Study Design and Objectives

This prospective, observational study was conducted at the *La Fe* University Hospital in Valencia, Spain. The study was performed in accordance with the Declaration of Helsinki and approved by the Ethics Committee for Clinical Investigation by the *La Fe* Hospital.

The main objective of this study was to assess the effectiveness and safety of OAT management in patients using the SintromacWeb compared to OAT management in patients using the conventional control (management at the hematology department). Therefore, the TTR of the INR in both groups were compared.

Secondary objectives were to assess the percentage of patients with INR within the therapeutic range at the time of each visit, the proportion of INR values within and outside of the therapeutic range, and the extreme values indicative of under-anticoagulation (INR<1.5) or over-anticoagulation (INR>5.0).

The safety objective was to assess the presence of abnormal coagulation events such as the number and severity of thromboembolism and bleeding events during the follow-up period.

### SintromacWeb System Description

The SintromacWeb system for OAT telecontrol at home consists of two key elements: a point-of-care (POC) device for patients’ INR self-testing and software that allows online interaction with physicians.

The POC device used was the HemoSense INRatio (Philips Remote Cardiac Services, Windsor CT), which is a monitoring system for INR home testing. The INRatio is capable of receiving the prothrombin time and INR results in less than a minute by using a small blood drop. A test strip is inserted into the INRatio monitor and a sample of fresh whole blood (15 µL) received from a finger prick is applied to the test strip. Blood is drawn into the test area by capillary action where it mixes with coagulation inducing reagents. The monitor performs the test and determines whether the controls are within pre-set limits.

**Figure 1:**
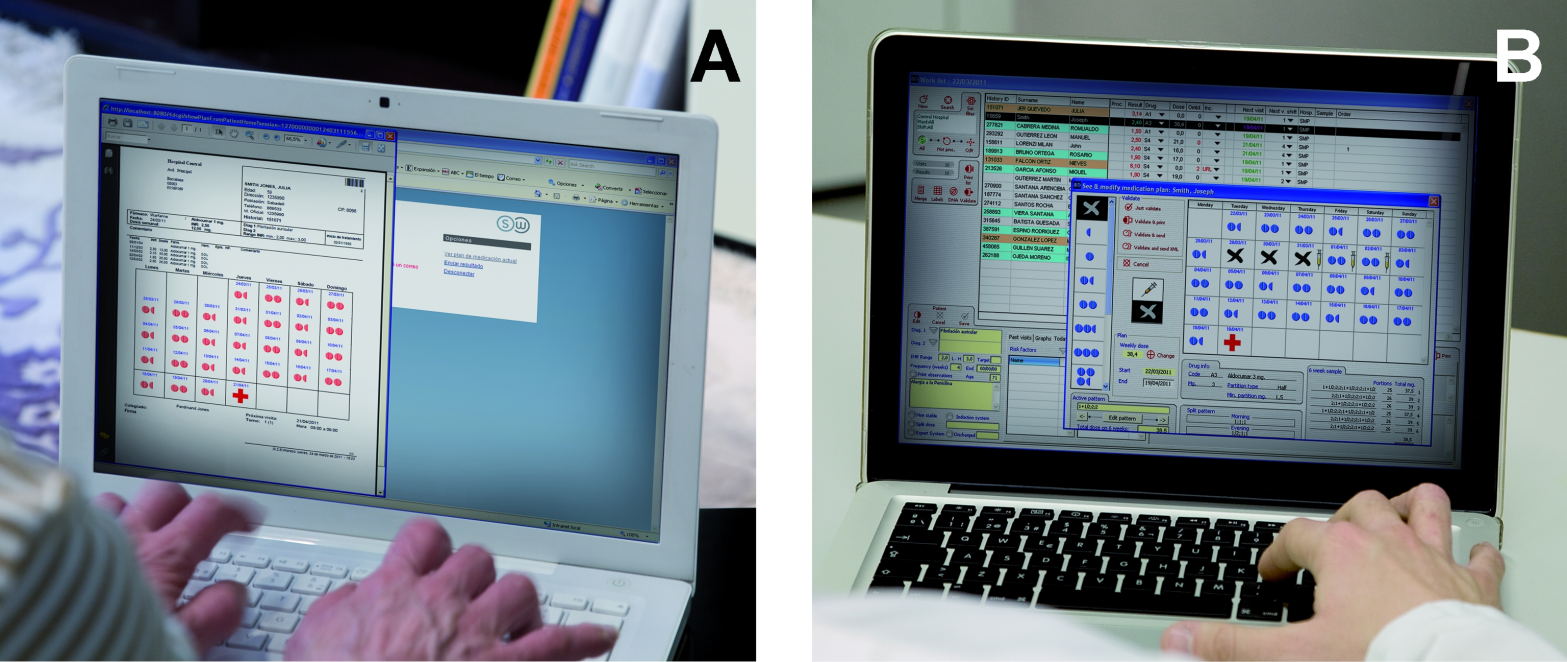
Illustrative pictures of the SintromacWeb software. In patient’s personal area (panel A), current medication schedule is available; INR results are introduced and sent to health care center. In physician’s area (panel B), the hematologist analyzes the data and introduces new medication schedule for patient.

The SintromacWeb software allows OAT patients to communicate with their doctors online, at home, or wherever an Internet access point is available. The SintromacWeb site is hosted by the same server as the *La Fe* Hospital website and is accessed through the hospital portal. Patients are provided with a username and password, which allows them to enter their personal area (Figure 1 A). The current medication schedule can be viewed, and according to the self-testing program, INR results are introduced and sent to the health care center. As a result, the hematologist can connect to the system, analyze the data, and introduce a new medication schedule for the patient (Figure 1 B). After the doctor has updated the schedule, an email is sent to the patient informing them that the treatment recommendations are available in their personal area of SintromacWeb.

### Patients and Recruitment

All patients were enrolled at the Hemostasis and Thrombosis Unit of the Hematology Service of the *La Fe* Hospital. The enrollment period lasted 3 months and patients were followed for 6 months.

The main inclusion criteria were: patients aged 18 years or older, under OAT treatment (either telecontrol or conventional control) for at least 3 months before inclusion in the study, and expected to be under OAT for at least 6 months after the inclusion in the study. The main exclusion criteria included patients who did not follow criteria for treatment adherence according to the investigator, patients who were not able to follow the visit schedule, and patients participating in another clinical trial during the study period.

Patients who met the criteria were identified by their physician when they came to the hematology department for a routine visit. Candidates were invited to participate in the study, and after acceptance, written informed consent was obtained. Patients were divided in two groups according to the OAT management that they were already following (conventional control or telecontrol with SintromacWeb). Patients using the telecontrol management system came from the cohort of the previous study in which the main inclusion criteria were patients aged 18 years or older with adequate technical facilities at home (computer and connection to the Internet) to run the Web-based telecontrol tool [21]. Recruitment ceased when the calculated minimal population size for each treatment group was reached.

### Variables of the Study

During the follow-up period, patients were required to visit the hematology department for OAT every time their physician considered it necessary, according to usual clinical practice. Variables of interest for the study were collected at four time points according to the following schedule: first visit (baseline) at the beginning of the study and at three follow-up visits (those closest to 2, 4, and 6 months after first visit). Variables corresponding to the 3 months prior to the first visit were obtained from the patient’s clinical records.

Information was gathered in a Case Report Form (CRF) specifically designed for the study. Confidentiality of the patient’s identity was preserved according to Spanish law for personal data protection.

Clinical data related to the study included starting date of OAT, indication for OAT, frequency scheduled by the physician for INR measurement, and target INR range and its changes during the follow-up period.

At the three follow-up visits, INR measurements and adverse events were collected. Adverse events included the presence of thromboembolism and its seriousness (major: deep thrombosis or non-transient ischemia; minor: surface thrombosis or transient ischemia ) as well as the presence of bleeding/hemorrhage and its seriousness (major: fatal and/or symptomatic bleeding into a critical area or organ, and/or bleeding that causes a decrease in hemoglobin ≥20 g/L, or which requires the transfusion of two or more units of whole blood or red blood cells, and/or gastrointestinal, urogenital, hemoptysis; minor: other not included in the described concepts).

### Time in Therapeutic Range (TTR) Assessments

For the purpose of this study, two therapeutic INR target ranges were defined: (1) INR 2-3 for OAT indications of deep vein thrombosis/pulmonary embolism, atrial fibrillation, heart valve disease, and heart valve bioprosthesis, and (2) INR 2.5-3.5 for indications of mechanical prosthetic heart valve, and venous thrombosis or stroke repetition in the context of antiphospholipid syndrome [22,23].

The primary endpoint of the study, TTR, was calculated using the approach proposed by Rosendaal [4], in which it is assumed that the INR value between two consecutive determinations varies linearly and the value of INR estimated for each of the days between consecutive measurements at intervals of increase/decrease of INR is ≥0.1. The Rosendaal method INR-specific for person-time takes into account the frequency of INR determinations and their actual values. By considering the individual values of INR collected at each visit with respect to the previous visit and the time elapsed between the two determinations, the TTR was calculated as the percentage of days within the therapeutic range out of the total days of treatment.

### Statistical Analysis

Population sample size was calculated by taking into consideration that the estimated average TTR is approximately 65% with a standard deviation (SD) of 20. Calculations showed that 174 patients (87 patients in each group) were needed to detect a difference of 8.5% in TTR between the two groups of OAT control (to meet the 5-10% difference between methods as recommended in the literature [6-8]) with a power of 80% and a significance value of *P*=.05. The analysis was stratified according to the type of OAT management to allow for the comparison between groups.

Continuous variables are presented as the mean (SD) or as median and range (minimum-maximum). For categorical variables, the number and percentage by category are used. For continuous variables, study groups were compared using Student’s *t* test. In case of different baseline characteristics between groups, comparison was made by adjusting variables using a linear regression model. For categorical variables, comparison between study groups was performed using the chi-square or Fisher’s test when the expected values of at least 80% of the cells in a contingency table were >5.

In all cases, statistical significance level was set at *P*≤.05. The statistical package SAS v. 9.2 for Microsoft Windows was used for calculations.

## Results

A total of 175 patients who met the inclusion criteria were enrolled in the study (88 in the conventional OAT control group and 87 in the telecontrol group). Two patients died before 3 months of follow-up: one in the conventional control group (an 84-year old female with obesity, diabetes, dyslipidemia, hypertension, and heart failure; death after aortic valve prosthesis implant) and one in the telecontrol group (a 57-year old male with obesity, dyslipidemia, hypothyroidism, heart failure, smoking habit, and chronic obstructive pulmonary disease; death from sudden cardiac arrest with anoxic encephalopathy).

**Figure 2:**
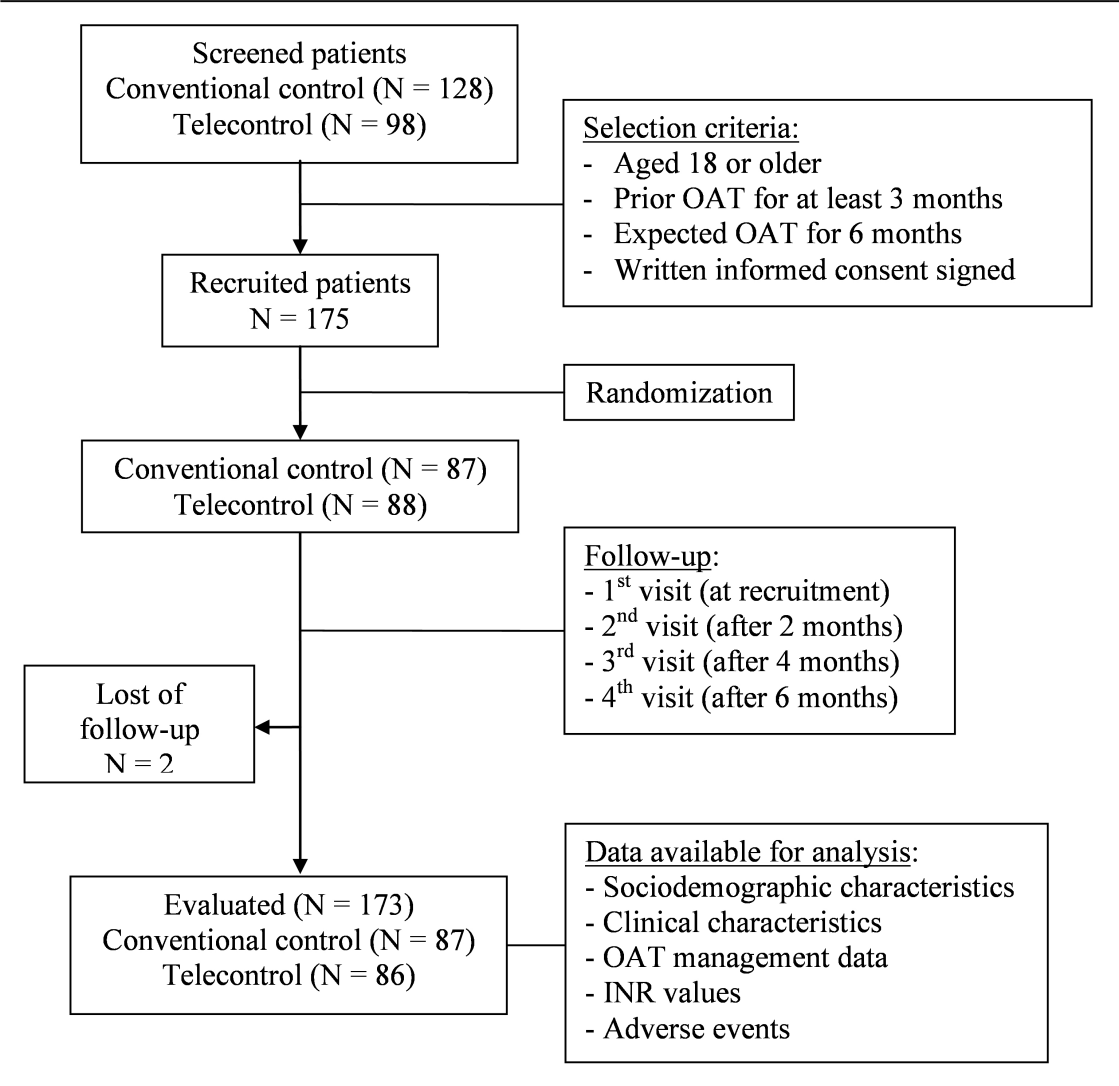
Flow of patients through the study.

At the end of the study, 173 patients were evaluated: 87 in the conventional control group and 86 in the telecontrol group. Follow-up time was a median of 6.3 (range 5.2-8.1) months with 6.4 (range 5.7-8.1) months in the conventional control group and 6.2 (range 5.2-7.1) months in the telecontrol group. Figure 2 shows the flow of patients through the study.

### Population Characteristics

The mean age of patients was 64.0 (SD 14.1) years (range 18-93) with 53.2% (92/173) of patients between 50 and 70 years. Patients were 50.3% (87/173) male and 95.4% (165/173) Caucasian. Most patients had a high school, college, or university education (58.4%, 101/173). Retirement was the most common labor status (50.6%, 87/172), while living accompanied (partner and/or with family: 86.7%, 150/173) was the most common family environment. Details of the sociocultural and environmental profile of patients in the two treatment groups are summarized in Table 1. Variables in both treatment groups were balanced with the exception of sex (more males in the telecontrol group) and family environment (more patients living alone in the conventional control group).

Most patients (97.7%, 169/173) presented comorbidities during the study. The most prevalent concomitant diseases were arterial hypertension (73.4%, 127/173), dyslipidemia (63.0%, 109/173), cardiovascular diseases (48.0%, 83/173), heart failure (34.1%, 59/173), obesity (34.1%, 59/173), diabetes mellitus (24.3%, 42/173), ischemic stroke (17.9%, 31/173), and chronic obstructive pulmonary disease (11.6%, 20/173). Hemorrhagic stroke, malignancy, inflammatory bowel disease, and nephritic syndrome were also present and had a prevalence <10%. Percentages of concomitant diseases were similar between the two treatment groups.

**Table 1:** Demographic, socio-cultural, and environmental profile of patients in the two oral anticoagulant therapy (OAT) management study groups: conventional control and telecontrol with the SintromacWeb system.

Variable		OAT management	
		Conventional (n=87)	Telecontrol (n=86)
		mean (SD) or n (%)	
**Demography**			
	Age, years, mean (SD)	65.0 (13.9)	62.9 (14.5)
	Body mass index, kg/m^2^, mean (SD)	27.4 (5.8)	27.5 (5.0)
	Male, n (%)	33 (38)	54 (63)^a^
	Caucasian, n (%)	83 (95)	82 (95)
**Educational level, n (%)**			
	No schooling	11 (13)	3 (4)
	Primary	32 (37)	26 (30)
	High School	20 (23)	24 (28)
	College / University	24 (28)	33 (38)
**Working status, n (%)**			
	Active worker	19 (22)	27 (31)
	Retired	45 (52)	42 (49)
	Housewife	14 (16)	8 (9)
	Temporary disability	2 (2)	2 (2)
	Unemployed	2 (2)	4 (5)
	Student	1 (1)	2 (2)
	Other	3 (4)	1 (1)
**Family environment, n (%)**			
	Living alone	17 (205)	4 (5)^b^
	Living with partner	49 (56)	55 (64)
	Living with relatives	20 (23)	25 (29)
	Other	1 (1)	2 (2)

^a^ *P*=.001

^b^ *P*=.04

### OAT Management Data

The most common OAT-requiring pathologies presented by patients were atrial fibrillation (65.3%, 113/173), cardiac valve prosthesis (38.7%, 67/173), and cardiac valvulopathy (37.6%, 65/173), while coronary artery disease, stroke, and deep vein thrombosis were presented by in around 15-20% of patients. Details of the OAT-requiring pathologies in the two treatment groups are summarized in Table 2.

The average time of OAT treatment prior to enrollment in the study was 9.2 (SD 6.4) years (8.1 years in the conventional control group and 10.2 years in the telecontrol group). Most patients were in the range of 5 to <10 years of treatment (31%, 27/87 in the conventional control group and 42%, 36/86 in the telecontrol group), followed by the range of 10 to <20 years (20%, 17/87 in the conventional control group and 33%, 28/86 in the telecontrol group), and the range of 1 to ˂5 years (31%, 27/87 in the conventional control group and 15%, 13/86 in the telecontrol group). A total of 14.4%, 25/173 of patients were <1 year or ≥20 years in treatment.

Median frequency of INR testing was higher in the telecontrol group (every 21 days; range 4-22) than in the conventional control group (every 35 days; range 14-45) (*P*<.001). The target INR ranges were virtually the same in both OAT groups: INR 2-3 in 56% and 57% (49/87 and 49/86, respectively) of patients and INR 2.5-3.5 in 44% and 43% (38/87 and 37/86, respectively) of patients. The INR target ranges did not change during the follow-up period.

**Table 2:** Oral anticoagulant therapy (OAT)-requiring pathologies of patients in the two study groups (conventional control and telecontrol with the SintromacWeb system).

Pathology	OAT management	
	Conventional (n=87)	Telecontrol (n=86)
	n (%)	
Atrial fibrillation	61 (70)	52 (61)
Cardiac valve prosthesis	34 (39)	33 (38)
Cardiac valvulopathy	36 (41)	29 (34)
Stroke of cardiac origin	20 (23)	16 (19)
Coronary artery disease	19 (22)	17 (20)
Deep venous thrombosis	13 (15)	13 (15)
Venous/arterial thromboembolism	10 (12)	11 (13)
Prevention of recurrent thromboembolism	10 (12)	11 (13)
Recurrent venous thrombosis	8 (9)	10 (12)
Myocardiopathy	5 (6)	9 (11)
Pulmonary thromboembolism	3 (3)	5 (6)
Other causes	35 (40)	36 (42)

### OAT Control Effectiveness

The mean TTR of INR—the primary endpoint of the study—was 8% higher (95% CI: 1.82-13.86) in the telecontrol group than in the conventional control group (62%, SD 21% vs 54%, SD 19%, respectively; *P*=.01). This difference represents a relative increase of 12.6%. The distribution of patients according to ranges of TTR is shown in Table 3. In absolute terms, TTR in the telecontrol group was 107 (SD 37) days while in the conventional control group TTR was 94 (SD 37) days (*P*=.02).

According to visits, TTR in the telecontrol group was always higher than in the conventional control group—the maximal difference being observed at the third visit (59% vs. 48%, respectively; *P*=.01).

**Figure 3:**
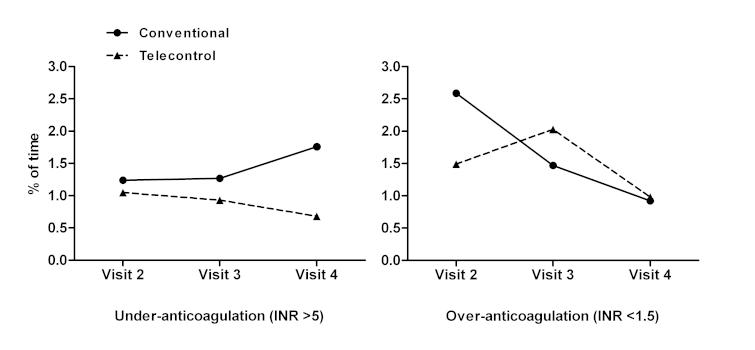
Evolution of average time in under- and over-anticoagulation (International Normalized Ratio [INR] <1.5 and INR>5, respectively) in the 3 visits of the follow-up period. The two oral anticoagulant therapy (OAT) management groups (conventional control and telecontrol with SintromacWeb system) are shown.

The average minimum and maximum INR values were 1.8 (SD 0.4) and 4.2 (SD 1.1), respectively. Only 19.7% (34/173) patients showed under-anticoagulation (INR<1.5) at some visit while over-anticoagulation (INR>5) was present in 22.0% (38/173) patients. Values were similar in the two OAT management groups. Figure 3 illustrates the evolution of the percentage of time in under- and over-anticoagulation during the 6-month follow-up. The mean percentage of time in which INR was <1.5 or >5 was 1.1% (SD 3.4%) and 1.4% (SD 3.6%), respectively.

**Table 3:** Distribution of patients of the two oral anticoagulant therapy (OAT) treatment groups (conventional control and telecontrol with the SintromacWeb system) according to ranges of percentage of time in therapeutic range (TTR) during the follow-up period.

TTR	OAT management	
	Conventional (n=87)	Telecontrol (n=86)
	n (%)	
<30%	9 (10)	8 (9)
30% – <50%	29 (33)	16 (19)
50% – <70%	31 (36)	29 (34)
≥70%	18 (21)	33 (38)

### OAT Control Safety

A total of 19 thrombotic and/or bleeding events during the follow-up period occurred in 13% (11/87) patients of the conventional control group, but only in 4% (3/86) patients of the telecontrol group (*P*=.03). The distribution of the events according to seriousness is shown in Table 4. Globally, 7 events were serious, 12 were non-serious, and most of them (5 and 10, respectively) occurred in the conventional control group.

The most common thrombotic event was transient ischemic attack (2 episodes), while there were single episodes of pulmonary thromboembolism, deep vein thrombosis, superficial venous thrombosis, and ischemic heart disease. Conversely, unspecified minor bleeding (9 episodes) and unspecified major bleeding (2 episodes) were the most common bleeding/hemorrhagic events. There was also one episode of gastrointestinal bleeding and one episode of urogenital bleeding.

**Table 4:** Distribution of thrombotic and bleeding events in the two groups of oral anticoagulant therapy (OAT) management groups (conventional control and telecontrol with the SintromacWeb system), according to seriousness.

Adverse events		OAT management	
		Conventional	Telecontrol
**Serious**			
	Thrombotic	3	0
	Bleeding	2	2
Total serious		5	2
**Non-serious**			
	Thrombotic	2	1
	Bleeding	8	1
Total non-serious		10	2
Total adverse events		15	4

## Discussion

### Principal Findings

Vitamin K antagonists are still the anticoagulant of choice for many patients, even in the face of the pharmacologic advantages shown by new oral anticoagulants targeting either thrombin or factor Xa. The higher drug cost of new oral anticoagulants often restricts their use to patients with risk of failure to maintain the INR in the therapeutic range (eg, with atrial fibrillation at risk for stroke). In addition, vitamin K antagonists are preferred for patients with uncertainties for dosing, such as those with renal dysfunction and those in marked extremes of body weight [24]. Therefore, the development of OAT self-testing and self-management systems to improve patient’s quality of life is warranted [6-9,14-20,25-27]. However, widespread implementation of computer-based, Internet-assisted systems within health care centers has not happened. This is the first study in which a previously described, validated, fully operative and currently in use telecontrol system in a hospital for OAT management at home (SintromacWeb) has been assessed in a prospective trial. Our results indicate that OAT management with the SintromacWeb is effective and safe in terms of clinical practice.

The observational nature of this study can be considered a limitation when interpreting the results. Investigators did not modify their usual clinical practice. In addition, since patients in the telecontrol group were recruited under defined clinical criteria as described in the previous study [21], some differences between the treatment groups were not unexpected. Two patient demographic variables were dissimilar between groups: the proportion of male/female and the proportion of patients living alone/accompanied. This is possibly linked to the fact that patients using the telecontrol tool were required to have a computer and a connection to the Internet at home [21]. The aim of the study was not to determine which OAT management system was superior, but to determine whether the telecontrol system is effective and safe in those patients who are able to use it based on clinical criteria in reference to patients using the conventional system, regardless of the patient profile in each group. In addition, the analysis was stratified to allow the comparison between groups.

Overall, the primary characteristics of the patients in our study were typical as a target of OAT treatment and within the clinic population values and ranges shown by other trials, such as mean age (64 years; range 57-75 in other trials) and percentage of males (50%; range 43-72% in other trials) [8,14-20]. The follow-up period of 6 months is also similar to most of those trials.

Patients in the telecontrol group achieved a relative 12.6% greater TTR value than patients in the conventional control group. Moreover, longer TTR in the telecontrol patients was consistently observed in all the scheduled visits during the study follow-up. Therefore, patients using the telecontrol system can be considered to have a good control of their OAT management. In some randomized trials, INR self-testing has been reported to be as good as [14,16,26] or better [8,15,17-20,25,27] than conventional testing. In particular, in a study performed with an Internet-based system for the supervised remote management of patients on OAT, Ryan et al indicated a TTR improvement of 15.4% in patients on INR self-testing [8]. However, since our study was observational, any comparison must be viewed with caution.

In our study, the frequency of INR measurements was higher in patients of the telecontrol group than in patients of the conventional control group (around 3 weeks vs 5 weeks, respectively). Although there is no consensus between the major guidelines on the optimal frequency of INR testing to achieve good INR control, it has been described that self-testing patients have increased frequency of INR measurements [28,29], which can be related to an improvement of the clinical outcomes of OAT.

In contrast with another trial [8], our study showed that both under-anticoagulation (INR <1.5) and over-anticoagulation (INR >5.0) occurred equally frequently in both the telecontrol and the conventional control groups. Our study, however, was not powered to detect differences under this approach.

Safety results indicated that patients in the telecontrol group showed significantly fewer adverse events than patients in the conventional control group. Similar results have been described in other trials [15,17,18], and other studies have demonstrated that the number of complications increases in parallel with the time that patients are outside therapeutic INR target range and with the occurrence of serious under- and over-anticoagulation [13,30,31]. Nevertheless, some other trials have shown that OAT self-control is as safe as conventional control [8,14,16,19,20].

### Conclusion

In conclusion, results of this study indicate that OAT management with the Internet-based telecontrol tool SintromacWeb is an effective and safe management system for those patients who are able to use it, based on clinical criteria. SintromacWeb is a system that has been previously validated, and it is fully operative and currently in use in a hospital, which adds value to the applicability of the study results to OAT patients eligible for telecontrol management.

## Abbreviations

CRF: case report form
INR: International Normalized Ratio
OAT: oral anticoagulation therapy
POC: point-of-care
TTR: time in therapeutic range

## References

[1] Heneghan Carl, Ward Alison, Perera Rafael, Bankhead Clare, Fuller Alice, Stevens Richard, Bradford Kairen, Tyndel Sally, Alonso-Coello Pablo, Ansell Jack, Beyth Rebecca, Bernardo Artur, Christensen Thomas Decker, Cromheecke M E, Edson Robert G, Fitzmaurice David, Gadisseur Alain P A, Garcia-Alamino Josep M, Gardiner Chris, Hasenkam J Michael, Jacobson Alan, Kaatz Scott, Kamali Farhad, Khan Tayyaba Irfan, Knight Eve, Körtke Heinrich, Levi Marcel, Matchar David, Menéndez-Jándula Bárbara, Rakovac Ivo, Schaefer Christian, Siebenhofer Andrea, Souto Juan Carlos, Sunderji Rubina, Gin Kenneth, Shalansky Karen, Völler Heinz, Wagner Otto, Zittermann Armin, Self-Monitoring Trialist Collaboration (2012). Self-monitoring of oral anticoagulation: systematic review and meta-analysis of individual patient data. Lancet.

[2] Navarro José L, Cesar Jesús M, Fernández María A, Fontcuberta Jordi, Reverter Joan C, Gol-Freixa Jordi (2007). [Morbidity and mortality in patients treated with oral anticoagulants]. Rev Esp Cardiol.

[3] Filippi Alessandro, Sessa Emiliano, Trifirò Gianluca, Mazzaglia Giampiero, Pecchioli Serena, Caputi Achille P, Cricelli Claudio (2004). Oral anticoagulant therapy in Italy: prescribing prevalence and clinical reasons. Pharmacol Res.

[4] Rosendaal F R, Cannegieter S C, van der Meer F J, Briët E (1993). A method to determine the optimal intensity of oral anticoagulant therapy. Thromb Haemost.

[5] Nichols W L, Bowie E J (1993). Standardization of the prothrombin time for monitoring orally administered anticoagulant therapy with use of the international normalized ratio system. Mayo Clin Proc.

[6] Fitzmaurice D A, Murray E T, McCahon D, Holder R, Raftery J P, Hussain S, Sandhar H, Hobbs F D R (2005). Self management of oral anticoagulation: randomised trial. BMJ.

[7] Menéndez-Jándula Bárbara, Souto Juan Carlos, Oliver Arturo, Montserrat Isabel, Quintana Mireia, Gich Ignasi, Bonfill Xavier, Fontcuberta Jordi (2005). Comparing self-management of oral anticoagulant therapy with clinic management: a randomized trial. Ann Intern Med.

[8] Ryan F, Byrne S, O'Shea S (2009). Randomized controlled trial of supervised patient self-testing of warfarin therapy using an internet-based expert system. J Thromb Haemost.

[9] Sawicki P T (1999). A structured teaching and self-management program for patients receiving oral anticoagulation: a randomized controlled trial. Working Group for the Study of Patient Self-Management of Oral Anticoagulation. JAMA.

[10] Gadisseur A P A, Kaptein A A, Breukink-Engbers W G M, van der Meer F J M, Rosendaal F R (2004). Patient self-management of oral anticoagulant care vs. management by specialized anticoagulation clinics: positive effects on quality of life. J Thromb Haemost.

[11] Regier Dean A, Sunderji Rubina, Lynd Larry D, Gin Kenneth, Marra Carlo A (2006). Cost-effectiveness of self-managed versus physician-managed oral anticoagulation therapy. CMAJ.

[12] McCahon Deborah, Murray Ellen T, Murray Kathryn, Holder Roger L, Fitzmaurice David A (2011). Does self-management of oral anticoagulation therapy improve quality of life and anxiety?. Fam Pract.

[13] Samsa G P, Matchar D B (2000). Relationship between test frequency and outcomes of anticoagulation: a literature review and commentary with implications for the design of randomized trials of patient self-management. J Thromb Thrombolysis.

[14] Dauphin Claire, Legault Benoît, Jaffeux Patricia, Motreff Pascal, Azarnoush Kasra, Joly Hélène, Geoffroy Etienne, Aublet-Cuvelier Bruno, Camilleri Lionel, Lusson Jean-René, Cassagnes Jean, de Riberolles Charles (2008). Comparison of INR stability between self-monitoring and standard laboratory method: preliminary results of a prospective study in 67 mechanical heart valve patients. Arch Cardiovasc Dis.

[15] Beyth R J, Quinn L, Landefeld C S (2000). A multicomponent intervention to prevent major bleeding complications in older patients receiving warfarin. A randomized, controlled trial. Ann Intern Med.

[16] Fitzmaurice D A, Hobbs F D, Murray E T, Holder R L, Allan T F, Rose P E (2000). Oral anticoagulation management in primary care with the use of computerized decision support and near-patient testing: a randomized, controlled trial. Arch Intern Med.

[17] Poller L, Keown M, Ibrahim S, Lowe G, Moia M, Turpie A G, Roberts C, van den Besselaar A M H P, van der Meer F J M, Tripodi A, Palareti G, Shiach C, Bryan S, Samama M, Burgess-Wilson M, Heagerty A, Maccallum P, Wright D, Jespersen J (2008). An international multicenter randomized study of computer-assisted oral anticoagulant dosage vs. medical staff dosage. J Thromb Haemost.

[18] Poller Leon, Keown Michelle, Ibrahim Saied, Lowe Gordon, Moia Marco, Turpie Alexander G, Roberts Christopher, van den Besselaar Aton M H P, van der Meer Felix J M, Tripodi Armando, Palareti Gualtiero, Jespersen Jørgen (2008). A multicentre randomised clinical endpoint study of PARMA 5 computer-assisted oral anticoagulant dosage. Br J Haematol.

[19] Claes Neree, Buntinx Frank, Vijgen Johan, Arnout Jef, Vermylen Jos, Fieuws Steffen, Van Loon Herman (2005). The Belgian Improvement Study on Oral Anticoagulation Therapy: a randomized clinical trial. Eur Heart J.

[20] Khan Tayyaba Irfan, Kamali Farhad, Kesteven Patrick, Avery Peter, Wynne Hilary (2004). The value of education and self-monitoring in the management of warfarin therapy in older patients with unstable control of anticoagulation. Br J Haematol.

[21] Ferrando Fernando, Mira Yolanda, Contreras María Teresa, Aguado Cristina, Aznar José Antonio (2010). Implementation of SintromacWeb(R), a new internet-based tool for oral anticoagulation therapy telecontrol: Study on system consistency and patient satisfaction. Thromb Haemost.

[22] Ansell Jack, Hirsh Jack, Hylek Elaine, Jacobson Alan, Crowther Mark, Palareti Gualtiero, American College of Chest Physicians (2008). Pharmacology and management of the vitamin K antagonists: American College of Chest Physicians Evidence-Based Clinical Practice Guidelines (8th Edition). Chest.

[23] Salem Deeb N, O'Gara Patrick T, Madias Christopher, Pauker Stephen G, American College of Chest Physicians (2008). Valvular and structural heart disease: American College of Chest Physicians Evidence-Based Clinical Practice Guidelines (8th Edition). Chest.

[24] Bauer Kenneth A (2013). Pros and cons of new oral anticoagulants. Hematology Am Soc Hematol Educ Program.

[25] Bereznicki Luke Ryan Elliot, Jackson Shane Leigh, Peterson Gregory Mark (2013). Supervised patient self-testing of warfarin therapy using an online system. J Med Internet Res.

[26] Harper P, Pollock D (2011). Improved anticoagulant control in patients using home international normalized ratio testing and decision support provided through the Internet. Intern Med J.

[27] O'Shea Susan I, Arcasoy Murat O, Samsa Gregory, Cummings Sandra E, Thames Elizabeth H, Surwit Richard S, Ortel Thomas L (2008). Direct-to-patient expert system and home INR monitoring improves control of oral anticoagulation. J Thromb Thrombolysis.

[28] Wells Philip S, Brown Allan, Jaffey James, McGahan Lynda, Poon Man-Chiu, Cimon Karen (2007). Safety and effectiveness of point-of-care monitoring devices in patients on oral anticoagulant therapy: a meta-analysis. Open Med.

[29] Ryan Fiona, O'Shea Susan, Byrne Stephen (2010). The 'carry-over' effects of patient self-testing: positive effects on usual care management by an anticoagulation management service. Thromb Res.

[30] White Harvey D, Gruber Michael, Feyzi Jan, Kaatz Scott, Tse Hung-Fat, Husted Steen, Albers Gregory W (2007). Comparison of outcomes among patients randomized to warfarin therapy according to anticoagulant control: results from SPORTIF III and V. Arch Intern Med.

[31] Oake Natalie, Fergusson Dean A, Forster Alan J, van Walraven Carl (2007). Frequency of adverse events in patients with poor anticoagulation: a meta-analysis. CMAJ.

